# MINIMALLY INVASIVE LAPAROSCOPIC ESOPHAGECTOMY VS. TRANSHIATAL OPEN ESOPHAGECTOMY IN ACHALASIA: A RANDOMIZED STUDY

**DOI:** 10.1590/0102-672020180001e1382

**Published:** 2018-08-16

**Authors:** Alberto Jorge Albuquerque FONTAN, João BATISTA-NETO, Ana Carolina Pastl PONTES, Marcos da Costa NEPOMUCENO, Tadeu Gusmão MURITIBA, Rômulo da Silva FURTADO

**Affiliations:** 1 Group of Esophageal, Stomach, Duodenum and Bariatric Surgery, Service of Digestive Surgery, University Hospital Prof. Alberto Antunes, Faculty of Medicine, Federal University of Alagoas, Maceió, AL, Brazil.

**Keywords:** Esophagectomy, Esophageal achalasia, Megaesophagus, Laparoscopy, Gastroparesis, Gastric stasis, Esofagectomia, Acalásia esofágica, Megaesôfago, Laparoscopia, Gastroparesia, Estase gástrica

## Abstract

***Background*:**

Open and laparoscopic trans-hiatal esophagectomy has been successfully performed in the treatment of megaesophagus. However, there are no randomized studies to differentiate them in their results.

***Aim*:**

To compare the results of minimally invasive laparoscopic esophagectomy (EMIL) vs. open trans-hiatal esophagectomy (ETHA) in advanced megaesophagus.

***Method*:**

A total of 30 patients were randomized, 15 of them in each group - EMIL and ETHA. The studied variables were dysphagia score before and after the operation at 24-months follow-up; pain score in the immediate postoperative period and at hospital discharge; complications of the procedure, comparing each group. Were also studied: surgical time in minutes, transfusion of blood products, length of hospital stay, mortality and follow-up time.

***Results*:**

ETHA group comprised eight men and seven women; in the EMIL group, four women and 11 men. The median age in the ETHA group was 47.2 (29-68) years, and in the EMIL group of 44.13 (20-67) years. Mean follow-up time was 33 months, with one death in each group, both by fatal aspiration. There was no statistically significant difference between the EMIL vs. ETHA scores for dysphagia, pain and in-hospital complications. The same was true for surgical time, transfusion of blood products and hospital stay.

***Conclusion*:**

There was no difference between EMIL and ETHA in all the studied variables, thus allowing them to be considered equivalent.

## RESUMO

**Racional:**

A esofagectomia trans-hiatal aberta e laparoscópica têm sido realizadas com êxito no tratamento do megaesôfago. Porém, não há estudos randomizados para diferenciá-las em seus resultados.

**Objetivo:**

Comparar os resultados da esofagectomia minimamente invasiva laparoscópica (EMIL) vs. esofagectomia trans-hiatal aberta (ETHA) no megaesôfago avançado.

**Método:**

Foram randomizados 30 pacientes, sendo alocados 15 em cada grupo - EMIL e ETHA. As variáveis estudadas foram escore de disfagia antes e após a operação no seguimento de 24 meses; escore de dor no pós-operatório imediato e na alta hospitalar; complicações do procedimento, comparando cada grupo. Foram também estudados: tempo cirúrgico em minutos, transfusão de hemoderivados, tempo de permanência hospitalar, mortalidade e tempo de seguimento.

**Resultados:**

Foram no grupo ETHA, oito homens e sete mulheres; no grupo EMIL, quatro mulheres e 11 homens. Faixa etária mediana no grupo ETHA foi de 47,2 (29-68) anos, e no grupo EMIL de 44,13 (20-67) anos. Tempo de seguimento médio foi de 33 meses, com um óbito em cada grupo, ambos por aspiração fatal. Não houve diferença estatística significativa, entre os grupos EMIL vs. ETHA quanto aos escores de disfagia, dor e complicações intra-hospitalares. O mesmo se verificou, quanto ao tempo cirúrgico, transfusão de hemoderivados e estadia hospitalar.

**Conclusão:**

Não houve diferença entre a EMIL e a ETHA em todas as variáveis estudadas, permitindo assim considerá-las equivalentes.

## INTRODUCTION

Surgical treatment of advanced megaesophagus is controversial^16^. There is no consensus among surgeons on which technique should be indicated in the treatment of their advanced forms. The ones that offer better results in the resolution of dysphagia present higher morbidity and mortality, and those with lower, increase the rate of relapse of the clinical aspects, with possible new interventions^2^ ^,^ ^4^ ^,^ ^14^ ^,^ ^18^. Open-access trans-hiatal esophagectomy, as an option for the treatment of advanced megaesophagus, has been consolidated^6^ ^,^ ^14^ ^,^ ^21^. In the 1990s, surgical diseases were impacted by videolaparoscopic access, reducing morbidity and favoring early recovery. De Paula et al.^13^ were the first to apply video access in the advanced chagasic megaesophagus, followed by others^1^ ^,^ ^8^ ^,^ ^12^ ^,^ ^17^ ^,^ ^22^ demonstrating that it is feasible. There is no consensus as to whether the efficacy of the videolaparoscopic approach in the treatment of megaesophagus exceeds open access.

Thus, the objective of this study was to compare the results of minimally invasive laparoscopic esophagectomy (EMIL) vs. open trans-hiatal esophagectomy (ETHA) in advanced megaesophagus.

## METHOD

The project was approved by the Research Ethics Committee of the Federal University of Alagoas, protocol nº 012257 / 2006-59.

Forty-four patients with advanced megaesophagus (groups 3 and 4 of the classification of Rezende^24^) were eligible from 2007 to 2013. Thirteen were excluded because they did not adhere to the proposed treatment. Thirty were randomized, randomly allocated by lot 15 in group EMIL and another 15 in group ETHA. Inclusion criteria were adults, 18-70 years old, with advanced megaesophagus; were excluded those with recurrent megaesophagus, patients with previous laparotomy in the upper abdomen, the ones with difficult to control comorbidities, and patients with associated portal hypertension.

All had preoperative surgical risk assessment according to ASA (American Society of Anesthesiologists), using the following measurements: blood count, coagulogram, nutritional index, echocardiogram, total abdominal ultrasonography and viral markers for hepatitis B and C. Were searched for Chagas’ disease through at least two methods of measurement.

The operation was trans-hiatal esophagectomy with truncal vagotomy without pyloroplasty and with manual endolateral esophagogastric anastomosis.

 The technique was the same in both groups^6^ ^,^ ^14^.

The variables studied were: 1) clinical dysphagia by score dysphagia according to the classification of Brandt^9^ - referring to the frequency, severity and type of dysphagia before and after the operation in the 1^st^ and 24^th^ month: mild (0-5), moderate (6-10) and intense (11-16); 2) pain score by verbal scale in the immediate postoperative period and at hospital discharge; 3) incidence of complications of the procedure in the cervical, thoracic and abdominal areas; 4) surgical time in minutes, blood transfusion, length of hospital stay, mortality and follow-up time.

### Statistical analysis

The statistical tests applied were chi-square and non-parametric Friedman, with significance of p<0.05.

## RESULTS

The mean age was 47.2 years (29-68) in group A. Regarding group B, it was 44.13 years (20-67). The gender in group A was seven men and eight women and group B 11 men and four women. The mean follow-up time was 33 months (1-100).

The serological evaluation for Chagas’ disease was positive in 20 patients (66.6%), and in the others it was not concluded in two measurements. All had epidemiological disease history and previous contact with triatomine (*Triatoma infestans*).

The comparison of the techniques in the dysphagia score shows that the severity of the dysphagia before the operation was classified as a severe score in any of the groups (86.6-93.3%), and in the postoperative period, in one and 24 months follow-up, 13 (86.6%) of the EMIL group were in light score (0-5) points, practically without dysphagia. In the ETHA group, 14 patients (93.3) were in this same pattern. In other words, there was no statistical difference in the dysphagia between the groups, according to Friedmann’s non-parametric test, p> 0.05, Table 1. The same result occurred when comparing the accesses, laparoscopic vs. open by the same test, p> 0.05.

**TABLE 1:** Comparison of the dysphagia score before and after operation between the laparoscopic (EMIL) and open trans-hiatal access (ETHA) groups, in the 30^rd^-day and 24-month follow-up

Dysphagia score	Preoperative	Postoperative
(30 days)	EMIL ETHA n=15 n=15	EMIL ETHA n=15 n=15
Mild (0-5)	0 0	13 (86.6%) 14 (93.3%)
Moderate (6-10)	2 (13.4%) 1 (6.7%)	2 (13.4%) 1 (6.7%)
Severe (11-16)	13 (86.6%) 14 (93.3%)	0 0
(24 months)		
Mild (0-5)	0 0	15 (100.0%) 15 (100.0%)
Moderate (6-10)	2 (13.4%) 1 (6.7%)	0 0
Severe (11-16)	13 (86.6%) 14(93.3%)	0 0

Friedman, p>0,05 (NS)

Pain score comparison in both techniques showed that in the immediate postoperative period the intensity of pain was similar in both groups, with pain absent in 26% of the patients; mild (66%) and intense (6%) in the EMIL group. In the ETHA group it was mild (60%) and moderate (6%). No patient had unbearable pain. At hospital discharge 94% of the patients had no pain in the open group and 86% in the laparoscopic group.

**FIGURE 1:**
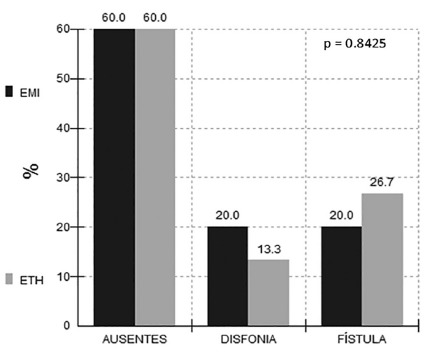
In-hospital complications of the two techniques in the cervical region, p> 0.05

Intra-hospital complications comparing the results in the two techniques in the cervical region were similar, and there was no statistical difference in the chi-square test, p> 0.05. There were no complications in 60% of patients in both groups. When present, transient dysphonia predominated in the EMIL group and cervical fistula in the ETHA group (Figure 1).

**FIGURE 2:**
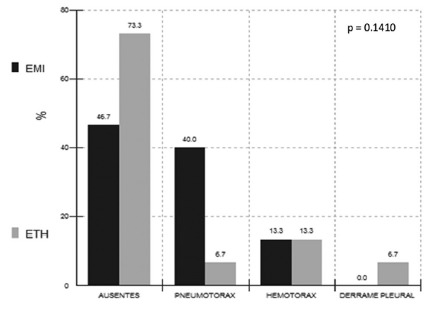
In-hospital complications of the two techniques in the thoracic region, p> 0.05

The in-hospital complications comparing the results in the thoracic region were also without statistical difference, p>0.05. However, the absence of complications was 73.3% in the laparoscopic group, and pneumothorax was more frequent than in the open group (Figure 2).

**FIGURE 3:**
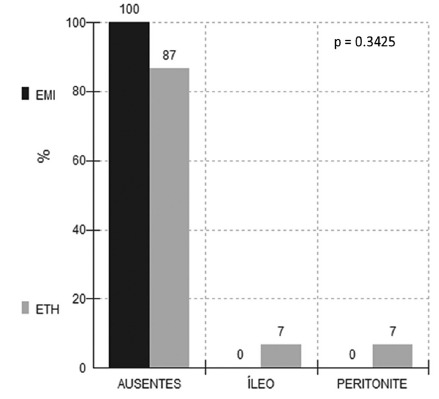
In-hospital complications of the two techniques in abdominal region, p>0.05

Complications, comparing the results in the two techniques in the abdominal region, also did not show statistical difference between one and the other access, p>0.05. In the open group there was one case of persistent ileus and one case of abdominal infection (abscess). No patient in the laparoscopic group had abdominal complication (Figure 3).

Regarding the length of hospital stay, it was lower in the ETHA group, mean of 14 days (7-17), but with no statistical difference. In group EMIL, the mean length was 17 days (5-28).

Regarding surgical time, the lowest was in the ETHA group, 120 min and in the EMIL, 180 min. The mean was 170 min for open access and 227 for laparoscopic.

No transfusion of blood products was required in any of the operated patients and there was a mortality rate of 6.7% (one case for each access route), and the cause of death was fatal aspiration in both.

Among the concomitant diseases four in the EMIL group presented schistosomiasis mansoni, chagasic cardiopathy, megacolon and gastroesophageal reflux disease, one disease for each patient. At ETHA, two had chagasic cardiomyopathy and one renal lithiasis. None of them had biliary lithiasis detected on total abdomen ultrasound.

## DISCUSSION

There is no consensus among surgeons^16^, which is the best technique for the treatment of advanced forms of megaesophagus^2^ ^,^ ^4^ ^,^ ^6^ ^,^ ^14^ ^,^ ^15^ ^,^ ^18^ ^,^ ^21^. Resection and cardioplasties, in their various techniques, are discussed among themselves. There are no randomized studies demonstrating superiority over one another^17^. The introduction of laparoscopic access into the surgical arsenal in the 1990s was so shocking that no one was able to serenely assess how far their limits and benefits would go^17^. Since then, the series in operations of high complexity began to be reported. De Paula et al.^13^, were the first to describe laparoscopic trans-hiatal esophagectomy. However, the acceptance of this technique by surgeons has been limited by the difficulty of visualizing the posterior mediastinum, laterally restricted work place, prolonged operative time and difficult learning curve^17^. Thus, to date, there are reported series^1^ ^,^ ^8^ ^,^ ^12^ ^,^ ^20^ ^,^ ^22^, but not randomized studies that clarify whether there is superiority of laparoscopic trans-hiatal access over open trans-hiatal. To our knowledge, this is the first prospective randomized trial to perform this assessment. Some series have suggested that minimally invasive laparoscopic esophagectomy is superior when compared to open access for trans-hiatal esophagectomy. The comparative series of case-control studies by Perry et al.^22^ concluded that mortality, blood loss, hospital stay, operative time and morbidity were no worse than in open access. In this study, mortality and morbidity did not find statistically significant results that indicated an advantage over one another method. There was one death in each group (6.7%), not linked to the operative method, but due to fatal aspiration due to gastric stasis, consequent to not performing pyloroplasty. Urschel et al.^27^ in meta-analysis had already warned that performing it or not, did not interfere in gastric stasis after truncal vagotomy in the transposed stomach. However, they pointed out that in the study performed there were two cases of aspiration and both were fatal. The same occurred in this study and the service adopted pyloroplasty in every transposed stomach^6^. Stasis appears even in those submitted to pyloroplasty, but afterwards it disappears^3^ ^,^ ^6^. The transposed stomach empties within the normal range, especially in orthostatic position. It acquires tubular form when it has normal emptying, and sacular proportional to the degree of stasis. Some degree of gastric atony may be found in the early postoperative period, attributed to vagotomy and dysphagia of the chagasic stomach, occasionally requiring the use of prokinetics, even though they are not very effective. In anterior series^6^ the tubular stomach was found in 32.1% (CI - 15.9-52.4%) and the saccular form in 10.7% (CI - 2.3-28.2%), therefore with stasis. In this randomized series it lasted for some patients from six months to two years to improve the clinical findings. In one case there was dilatation of the pylorus. In another after eight years of laparoscopic access, gastric stasis was still so important that it required hospitalization, nasogastric intubation, enteral nutritional and clinical handling. It should be noted that at the time it was believed that truncal vagotomy did not involve obligatory pyloroplasty, a topic still controversial and current, where the transposed stomach empties itself into normal patterns, especially if the patient is in orthostasis.

Another variable studied in this study was the pain score, whose results were better for the laparoscopic group, but with no statistical difference. Regarding efficacy in the resolution of dysphagia, analyzed according to criteria well determined by Brandt^9^, there was no superiority between laparoscopic or open methods. The same occurred in the morbidity, regarding the complications by regions and it was observed that in laparoscopy there are more sequelae of pneumothorax, but without statistical difference. Perhaps it could be explained by the pressure of the gas, which, while facilitating dissection, invades the structures more frequently.

In the comparison of the efficacy of EMIL vs. ETHA, there was no statistical advantage of one access over the other. However, for a definitive answer it is necessary to have multicentric studies with broader casuistics^17^ ^,^ ^20^, a limiting factor in this study. There is also epidemiological restriction to obtain expressive casuistics, due to the number of cases of achalasia/year in advanced degree being small, either by chagasic or idiopathic etiology.

Technically it is worth mentioning that in the case of videolaparoscopy cervical access can only be performed when the operation has advanced greatly in the mediastinum. Otherwise, the gas dissipates and makes it very difficult to follow the surgical procedure. Sometimes small emphysema occurs in the cervical region and the veins of the region become prominent.

No patient received transfusion of blood products; however, some were submitted to enteral or parenteral nutritional recovery to reach the preoperative minimum index of 18-20 BMI. As for the surgical time, the open operation was faster and the shortest time was 120 min (mean 170); in the laparoscopy it was 180 min (mean of 227). There was one death in each group related to gastric stasis due to the lack of pyloroplasty^23^ ^,^ ^26^. The fistula index (26%) found no difference between EMIL and ETHA and was similar to the literature (10-26%)^11^ ^,^ ^28^; was lower in laparoscopy (20%), but without statistical significance. With mechanical laterolateral esophagogastric anastomosis^21^ the rate of fistulas in the surgical service of the authors was reduced to 10-12%^7^; the same has been demonstrated by other authors^10^ ^,^ ^21^ ^,^ ^25^.

## CONCLUSION

There was no difference between laparoscopic minimally invasive trans-hiatal esophagectomy (EMIL) and open trans-hiatal esophagectomy (ETHA) in all studied variables, thus allowing to be considered equivalent.
